# Polymorphisms in Interleukin 13 Signaling and Interacting Genes Predict Advanced Fibrosis and Hepatocellular Carcinoma Development in Non-Alcoholic Steatohepatitis

**DOI:** 10.3390/biology9040075

**Published:** 2020-04-09

**Authors:** Marwa O. El-Derany

**Affiliations:** Biochemistry Department, Faculty of Pharmacy, Ain Shams University, Cairo 11566, Egypt; marwa.omar@pharma.asu.edu.eg

**Keywords:** NASH, HCC, IL-13, STAT6, YAP1, PD-L2

## Abstract

Background: non-alcoholic steatohepatitis (NASH) recently headlined hepatocellular carcinoma (HCC) worldwide. This study aims to unveil the role of some unaddressed critical players that might aid in understanding, predicting, and targeting NASH and NASH-HCC. Methods: Serum interleukin 13 (IL-13) levels and single nucleotide polymorphisms (SNPs) within interleukin (IL)-13 rs20541, IL-13 receptors (IL-13R1) rs2248841, (IL-13R2) rs5946040, signal transducer activator of transcription 6 (STAT6) rs167769, yes-associated protein (YAP1) rs11225163, programmed death-ligand 1 (PD-L1) rs2282055, and programmed death-ligand 2 (PD-L2) rs7854413 genes were analyzed by qRT-PCR. Multiple stepwise regression analysis was performed on a cohort of 134 Egyptian male patients diagnosed with NASH and NASH-HCC. RESULTS: higher serum alpha-fetoprotein (AFP) and higher serum IL-13 levels were directly associated with HCC development in NASH (odds ratio (OR) 19.6 and 1.9 *p* < 0.01). Reversibly, the presence of the C/C genotype in STAT6 rs167769 and the C allele carrier YAP1 rs11225163 were inversely associated with HCC in NASH patients (OR 0.015 and 0.047 *p* < 0.01). A predictive model was formulated with 97.5% specificity, 90.9% sensitivity, and 94.8% accuracy. Moreover, higher serum IL-13 levels and the presence of PD-L2 rs7854413 C allele carriers were associated with advanced fibrosis progression in NASH patients (OR 1.432 and 3.797 *p* < 0.01). Serum levels of IL-13 and C/C genotype in STAT6 rs167769 significantly increased the predictive capacity of serum AFP to predict HCC in F1–F2 and in F3–F4 fibrosis grades NASH patients. Conclusion: association between serum IL-13 and PD-L2 rs7854413 polymorphism successfully predict advanced fibrosis in NASH. However, HCC development in NASH is associated with higher serum AFP, IL-13 levels, and STAT6 rs167769, YAP1 rs11225163 polymorphisms.

## 1. Introduction

Despite increasing awareness against obesity, it is still rising dramatically, precipitating multiple complications. Nowadays, non-alcoholic fatty liver disease (NAFLD), hepatic manifestation of hyperlipidemia, is considered the most common form of chronic liver disease [1]. Non-alcoholic fatty liver (NAFL) is a complex metabolic disease that starts with hepatic lipid accumulation. This aggravates liver inflammation (which can lead to hepatocyte injury and death), and one can end up with pericellular fibrosis. At this point, it turns into a more aggressive form termed non-alcoholic steatohepatitis (NASH) [2]. Thus, NASH is recognized as a multidisciplinary disease that has evolved from metabolic, inflammatory, and immune derangements. The progression of cirrhosis and hepatocellular carcinoma (HCC) from NASH is dramatically increasing, marking NAFLD as the second leading indication for liver transplantation [3].

A great deal of research has focused on NAFLD molecular and immunological mechanisms that derives fibrosis, cirrhosis and HCC progression [4]. However, understanding the mechanisms of fibrogenesis in NASH could reveal novel drug targets, as well as novel predictors that might have a major impact on morbidity and mortality. It is now clear that profound immune and inflammatory dysregulation in chronic obesity is intricately associated with NASH progression. In fact, type 1 inflammation is considered the major hallmark of chronic obesity. However, recent researches highlighted the role of type 2 immunity in the disease progression as well [5]. Despite being engaged in critical protective activity in metabolic diseases, by reducing tissue inflammation and activating important tissue-regenerative mechanisms, type 2 cytokine-mediated repair processes that exacerbate the progression of fibrosis when chronically dysregulated or over-exuberant [6,7].

Type 2 immunity is characterized by increased production of signature cytokines interleukin (IL)-4, IL-5, IL-9, and IL-13 [8]. Of these, IL-13 has been identified as the dominant effector cytokine of liver fibrogenesis of different etiologies [9,10]. Specifically, serum IL-13 levels were reported as significantly increased in NASH patients. It plays a pleiotropic function in the hepatic response to metabolic stress. Besides, it was proven that IL-13 is overexpressed in activated hepatic stellate cells (HSCs) involved in NASH fibrosis progression [11]. More interestingly, a recent report found that co-inhibition of IL-13 and transforming growth factor-β (TGF-β) signaling attenuate the fibrotic machinery more completely than inhibiting TGF-β alone in NAFLD-associated fibrosis [6]. Additionally, a recent correlation of gut microbiota and HCC development in NAFLD was suggested to be through the upregulation of IL-13 [12]. 

Mechanistically, IL-13 binds to two known chain alpha-receptors, IL13Rα1 and IL13Rα2, which distinctively activate two signaling cascades, depending on the activated receptor hitherto mentioned. The first signaling axis is activated via the IL13Rα1 chain receptor. IL13Rα1 is a low-affinity receptor that forms a heterodimer with the IL-4Rα chain to form a high-affinity receptor (IL-13R). This high affinity receptor mediate signal transduction through the Janus kinase/signal transducer and activator of transcription (JAK-STAT)-6 pathway, which promotes liver fibrosis [13]. The second signaling axis is activated via IL13Rα2, a high-affinity receptor. It acts as a decoy receptor that signals in a signal transducer activator of transcription 6 (STAT6)-independent manner [14]. Studies showed that IL13Rα2 positively regulates HCC through activation of the TGF-β1 promoter, increasing TGF-β1 production and fibrosis progression [11,15]. Others suggested that it might mediate its effect via activating the Yes-associated protein (YAP) and/or transcriptional coactivator with PDZ-binding motif (TAZ) in hippo signaling [15,16]. Interestingly, the hippo pathway transcriptional activator TAZ was recently correlated in fibrosis progression in NASH [17]. However, little is known about the role of YAP in NASH pathogenesis and progression into cancer.

It is well established that inflammation derive fibrogenesis and cancer progression via imposing cell injury with aberrant healing processes. Interestingly, inflammation also contribute to the suppression of immunological surveillance mechanisms via altered expression of programmed death 1 (PD-1), programmed death-ligand 1 (PD-L1), and programmed death-ligand 2(PD-L2) [18]. Whereas, PD-1 has a high affinity for PD-L2 such that its binding is accompanied by the formation of a prominent pocket in human PD-1[19]. It was recently shown that IL-13 producing cells, type 2 innate lymphoid cells (ILC2s), are destabilized in response to PD-1/PD-L1 pathway upon high-fat feeding resulting in impaired tissue metabolism [20]. Furthermore, PD-1 blockade showed a partial restore in type 2 innate axis and could serve as promising targets of immune-modulatory NASH therapy [21,22,23,24,25]. Importantly, PD-L1 was found to be responsible for HCC development in NASH via inducing the exhaustion of HCC-directed CD8^+^ T cells [26]. Similarly, the interaction of PD-1 and PD-L1/ PD-L2 is generally correlated with immune tolerance and evasion in HCC [27]. Therefore, PD-L1 and PD-L2 might represent possible predictive candidates for fibrosis progression and cancer development in NASH.

Genetic variation in different individuals translates their individualized risk for disease susceptibility and progression. Genome-wide association studies (GWASs) have generally focused on identify single-nucleotide polymorphisms (SNPs) in different genes to predict disease susceptibility and outcomes [28]. Based on the aforementioned, investigating different SNPs in IL-13 gene, IL13Rα1 gene, IL13Rα2 gene, STAT6 gene, YAP gene, PD-L1, and PD-L2 genes with fibrosis progression and HCC development in NASH have not been studied yet. Accordingly, this study uniquely aims to underscore, for the first time, the influence of selected SNPs in fibrosis progression and HCC development in NASH Egyptian male patients.

## 2. Subjects and Methods

### 2.1. Study Population

One hundred and thirty-four well diagnosed male NASH and NASH-HCC Egyptian patients were enrolled in this study. They were recruited from a specialist, Dr. Yassin Abdel Ghaffar, from the Center for Liver Diseases and Researches in Cairo. The study was approved by the Research Ethics Committee, Faculty of Pharmacy, Ain Shams University, under memorandum no. ENREC-ASU-66. Informed consent was obtained from each patient for participation and publishing results. The study was carried out under the regulations and recommendations of the Declaration of Helsinki.

The population study included 55 male Egyptian Caucasian patients with primary HCC arising from a background of NASH. The diagnosis was determined through assessment according to the European Association for the Study of the Liver (EASL) guidelines and on histologic sampling [29]. The second group in this study included 79 male NASH patients. The diagnosis of NASH was established through radiological evidence of hepatic steatosis and on histological assessment when liver biopsy was available. NASH patients were sub-classified according to the degree of liver fibrosis: F1 and F2 as low fibrosis grade and F3 and F4 as high fibrosis grade. Histopathological data were collected when a liver biopsy was performed. Liver biopsy specimens were scored using the Meta-analysis of Histological Data in Viral Hepatitis (METAVIR) system [30] and the severity of steatosis was scored according to the validated Kleiner criteria [31]. All biopsies were evaluated and scored by the same pathologist.

Patients with alcohol intake, infected with viral hepatitis, diagnosed with an autoimmune disease, or subjected to drugs that induced liver injury were all excluded from the study. Baseline and clinical characteristics are summarized in Table 1. Basic anthropometric parameters as body mass index (BMI) was calculated.

### 2.2. Laboratory Analyses

Blood samples for hematological, biochemical analysis, and genomic DNA extraction were obtained after an overnight fast with the standard methods at the center’s laboratory. For each patient, three blood samples were taken. Two samples were collected on EDTA vacutainer for whole blood preparation and the third sample was collected on plain vacutainer for serum separation. Whole blood was used for hematological analysis and genomic DNA extraction. Routine hematological analysis was done for the whole blood, and it included a complete blood picture (CBC). Serum was separated by centrifugation of the plain vacutainer at 4000 rpm for 10 min at 4 °C and then aliquoted and stored at −80 °C for subsequent use in biochemical tests. These tests included fasting blood glucose (FBG), serum transaminases (alanine aminotransferase (ALT), aspartate aminotransferase (AST)), total bilirubin, serum albumin, serum triglycerides, serum insulin, serum alpha-fetoprotein (AFP), and serum IL-13 (documented in Table 1).

### 2.3. Genomic DNA Extraction

Genomic DNA was extracted with QIAamp DNA Mini Kit protocol (QIAGEN, Santa Clarita, CA, USA) according to the manufacturer’s instructions. DNA samples were subjected to DNA quantitation and purity assessment using the NanoDrop^®^ (ND)-1000 spectrophotometer (NanoDrop Technologies, Inc., Wilmington, DE, USA). 

### 2.4. Genotyping of Studied Genes

Genotyping was carried out by the use of quantitative real time polymerase chain reaction (qRT-PCR) reagents, constituting a ready-to-use system designed TaqMan assay to detect the following SNPs: IL-13, rs20541; IL-13R1, rs2248841; IL-13R2, rs5946040; STAT6, rs167769; YAP1, rs11225163; PD-L1, rs2282055; PD-L2, rs7854413 (custom TaqMan SNP assay C___2259921_20, C__15754956_10, C___9190226_10, C____620401_20, C__27134150_10, C___1409286_1_, C__27984834_10, respectively, using Assays-by-Design supplied by Applied Biosystems International ABI; Applied Biosystems, Foster City, CA, USA). The SNPs were also selected on the basis of allele frequencies and functional analysis as they were reported to cause either Mis-sense Mutation, Transversion, Transition Substitution, or Transversion Substitution at an untranslated region. Additionally, they were selected on their clinical implications [32,33] (website http://www.ncbi.nlm.nih.gov/projects/SNP/). Putative departures of the Hardy–Weinberg Equilibrium were calculated by using the software Haploview 4.1. [34].

### 2.5. Serum IL-13 and Serum Insulin Levels

Serum IL-13 was quantified using an enzyme-linked immunosorbent assay (ELISA) technique using a commercially available kit (Bioassay, Biotech, CO.; Ltd, Hangzhou, China). Serum insulin was also quantified using a commercially available ELISA kit (Nova Tec Immundiagnostica GmbH, Dietzenbach, Hessen, Germany). The homeostatic model assessment of insulin resistance (HOMA-IR) was calculated from fasting insulin and FBG by the following equation: HOMA-IR = fasting insulin (µU/mL) × FBG (mg/dL)/405 [35]. All ELISA procedures were done by Hyprep Automated ELISA system (Hyperion Inc., Miami, FL, USA) according to the manufacturer’s instructions.

### 2.6. Statistical Analysis

IBM SPSS statistics (V. 22.0, IBM Corp., Armonk, NY., USA, 2013) was used for data analysis. The Shapiro–Wilk test was used to test the normal distribution of data. Continuous parametric variables were expressed as mean ± SD, while non-parametric data were expressed as median (range). Additionally, categorical variables were presented as frequencies (percentage). A comparison between two independent parametric variables were performed using Student’s t-test, and Wilcoxon rank-sum test was carried out for comparison between two non-parametric variables. The Hardy–Weinberg equilibrium was assessed in the study population. A general linear model was used to control for potential confounders. Significant covariates at binary logistic regression analysis were included in a multivariate stepwise logistic regression model with a forward approach to identify independent predictors of fibrosis or cancer. Besides, any skewed data were logarithmically transformed before performing simple and multiple binary stepwise regression analyses. The area under the receiver operating characteristic curve (ROC) indicated the prediction capacity of this analysis. The results were reported as odds ratio (OR) and 95% confidence intervals (CIs). A difference of *p* < 0.05 was considered significant.

## 3. Results

### 3.1. Study Characteristics of NASH and NASH-HCC Patients

One hundred and thirty-four patients were recruited in this study. The clinical and demographic characteristics of NASH-HCC and NASH patients are provided in Table 1. Patients were classified as follows: 58.9% (*n* = 79) were classified as NASH and 41% (*n* = 55) were classified as NASH-HCC. The univariate regression analysis showed that patients with NASH-HCC were significantly older than NASH patients (OR = 1.073, 95% CI = 1.012–1.138, *p* = 0.017). Additionally, higher serum AFP levels and higher serum IL-13 levels were significantly associated with NASH-HCC at (OR = 7.641, 95% CI = 3.6–16.18, *p* < 0.001) and (OR = 1.616 95%, CI = 1.3–2.008, *p* < 0.001), respectively. This study showed significant difference between lower fibrosis grades F1–F2, advanced fibrosis grades F3–F4, and cirrhosis among NASH and NASH-HCC patients at *p* < 0.001. Whereas, 51.8% showed lower fibrosis grades F1–F2 in NASH and 48.1% showed advanced fibrosis grades F3–F4. However, cirrhosis was absent among NASH patients. On the other hand, 90.9% were confirmed to be cirrhotic among NASH-HCC patients and only 9% were of advanced fibrosis grade F3–F4, with complete absence of lower fibrosis grades, among NASH-HCC patients.

Regarding genes polymorphisms, the association between IL-13 rs20541; IL-13R1 rs2248841; IL-13R2 rs5946040; STAT6 rs167769; YAP1 rs11225163; PD-L1 rs2282055; PD-L2 rs7854413 with cancer development in NASH were shown in Table 2. The results explored that the presence of IL-13 rs20541 G/G genotype (chi^2^ = 10.593 *p* = 0.004 OR = 7.07 95% CI = 1.89–26.44), the presence of IL-13R2 rs5946040 T/T genotype (chi^2^ = 4.51 *p* = 0.038 OR = 2.656 95% CI = 1.056–6.677), the absence of STAT6 rs167769 C/C genotype (chi^2^ =21.6 *p* < 0.001 OR = 0.124 95% CI = 0.052–0.297), the absence of C allele carrier in YAP1 rs11225163 (chi^2^ = 5.56 *p* = 0.022 OR = 0.295 95% CI = 0.103–0.842), and the presence of PD-L1 rs2282055 T/T genotype (chi^2^ = 5.76 *p* = 0.022 OR = 3.7 95% CI = 1.206–11.352) were all significantly associated with the development of HCC in NASH at simple binary univariate regression analysis, with highest significance being recorded for STAT6 rs167769 C/C genotype.

### 3.2. Association of Adjusted Univariate Significant Parameters with HCC Development in NASH Patients

To determine whether the significant univariate parameters influence the development of HCC in NASH patients after adjustment of significant covariate as age. A general linear model was performed to control the confounding variable. The results in Table 3 showed that all adjusted values for serum AFP, serum IL-13, the presence of G/G genotype in IL-13 rs20541, the presence of T/T genotype in IL-13R2 rs5946040, the presence of T/T genotype in PD-L1 rs2282055, the absence of C/C genotype STAT6 rs167769, and the absence of C allele carrier in YAP1 rs11225163 were all still significantly associated to HCC development in NASH with *p* < 0.05.

### 3.3. IL-13/STAT6 Signaling Axis and YAP1 Are Critical Players for HCC Development in NASH Patients

This study aimed to formulate a predictive model using all adjusted significant univariate variables. Multiple stepwise logistic regression analysis was performed after controlling for significant confounder (age), as shown in Table 3, and showed that only higher serum AFP levels and higher serum IL-13 levels were found to be directly related to HCC development (OR = 19.6, *p* < 0.001) and (OR = 1.9, *p* = 0.005), respectively. On the other hand, the presence of the C/C genotype in STAT6 rs167769 and the carrier state of the C allele in YAP1 rs11225163 were inversely associated with HCC in NASH patients (OR = 0.015, *p* < 0.001) and (OR = 0.047, *p* = 0.004), respectively. A predictive equation predicting the probability for HCC development in NASH patients (P) was estimated with formula:

P = 1/1 + e^− (− 17.996 + 0.693 × a + 3.101 × b + 0.247 × c-4.472 × d − 3.316 × e)^

where the substitution values were as follow: **a**: serum IL-13 levels (ng/L), **b:** log serum AFP levels (ng/mL), **c**: age of the patients in years, **d**: STAT6 rs167769 C/C genotype carrier=1, non-carrier=0, **e:** YAP1 rs11225163 C allele carrier = 1, non-carrier = 0.

**Figure 1:**
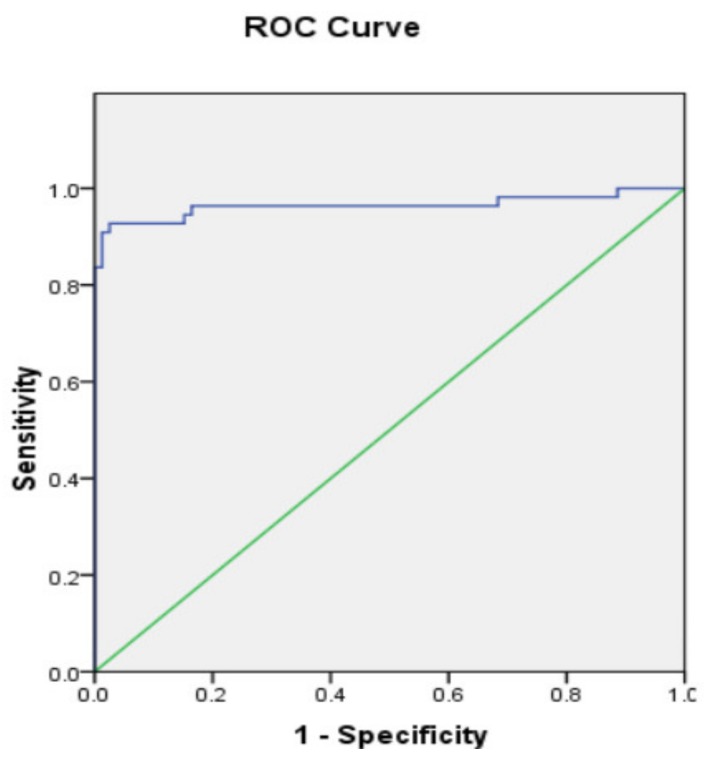
Receiving operating curve (ROC) of the multivariate analysis for HCC development in NASH patients. Area under the curve (AUC) was 0.964 (95% CI = 0.92–0.999).

The ROC was plotted in accordance with the same model as expressed in Figure 1. The predictive capacity of the final model was excellent as indicated by the area under the ROC curve 0.964 (95% CI = 0.92–0.999). Where the best cut-off value for this model was 0.511. This means that patients found above this cut-off value might experience HCC development on NASH background. Multiple stepwise logistic regression analysis showed that this model presented showed specificity of 97.5% and significantly increase the sensitivity of serum AFP levels from 60% to 90.9% as well as increase the accuracy of serum AFP levels from 83.6% to 94.8%.

### 3.4. Sub-Classifying NASH Patients According to Their Fibrosis Grades

Interestingly, this study investigated the association of different predictors with advanced fibrosis in NASH patients after excluding cancer patients. Whereas, 50.6% (n = 40) were of lower fibrosis grades F1–F2 and 49.3% (*n* = 39) were of advanced fibrosis grades F3–F4. Univariate analysis showed that higher serum IL-13 levels (OR = 1.496 95%, CI = 1.111–2.014, *p* = 0.008), the presence of IL-13 rs20541 A allele carrier state (chi^2^ = 4.617, *p* = 0.034, OR = 2.722 95%, CI = 1.081–6.6858), the absence of IL-13R2 rs5946040 G allele carrier state (chi^2^ = 6.388, *p* = 0.018, OR = 0.194, 95% CI = 0.05–0.756), and the presence of PD-L2 rs7854413 C allele carrier state (chi^2^ = 7.495, *p* = 0.001, OR = 5.5 95% CI = 1.963–115.411) were all associated with advanced fibrosis grades in NASH patients, as shown in Table 4.

### 3.5. IL-13/PD-L2 Are Crucial for Fibrosis Progression in NASH Patients

Multivariate binary logistic regression analysis was made, and found that older age is not associated with advanced fibrosis progression at *p* = 0.335. On the other hand, higher serum IL-13 levels and the presence of PD-L2 rs7854413, C allele carriers states are directly associated with advanced fibrosis progression in NASH patients (OR = 1.432 95%, CI = 1.022–2.008, *p* = 0.037) and (OR = 3.797, 95% CI = 1.216–11.875, *p* = 0.022), respectively, as shown in Table 5. The ROC was plotted in accordance with the same model showed that the area under the ROC curve was 0.783 (95% CI = 0.703–0.862) with 77.5% specificity, 72.2 % accuracy, and 66.7% sensitivity. 

### 3.6. Comparing Different Fibrosis Grades in NASH with HCC Development

Multiple multinomial logistic regression analysis was done after adjusting significant cofounder (age) to compare advanced fibrosis grades F3–F4 with HCC development in NASH, as well as to compare lower fibrosis grades F1–F2 with HCC development in NASH. It was found that by comparing advanced fibrosis grades F3–F4 with HCC-NASH, serum AFP level has a moderate predictive power, where the area under the ROC curve was 0.751 (95% CI = 0.651–0.851) with 76.9% specificity, 69.1% accuracy, and 63.6% sensitivity. Interestingly, this study found that higher serum IL-13 levels and higher serum AFP levels were associated with cancer (OR = 1.625, 95% CI = 1.077–2.451, *p* = 0.021) and (OR = 8.231, 95% CI = 2.668–25.394, *p* < 0.001) respectively. However, the presence of the C/C genotype in STAT6 rs167769 was inversely associated with HCC development, compared to advanced fibrosis grades F3–F4 (OR = 0.029, 95% CI = 0.004–0.197, *p* < 0.001). This model showed strong predictive capacity with the area under the ROC curve 0.936 (95% CI = 0.885–0.986) and 87.2% specificity, 88.3% accuracy, and 89.1% sensitivity.

Similarly, the association of HCC development with lower fibrosis grades F1–F2 in NASH patients showed, also, that serum AFP levels alone has moderate predictive power, where the area under the ROC curve was 0.769 (95% CI = 0.67–0.868) with 92.5% specificity, 75.8% accuracy, and 63.6% sensitivity. However, IL-13 and STAT6 significantly improved the prediction power for HCC development in F1 and F2 NASH patients. This study showed that higher serum IL-13 levels and higher serum AFP levels were directly associated with cancer (OR = 2.036, 95% CI = 1.384–3.076, *p* = 0.001) and (OR = 9.151, 95% CI = 2.94–28.425, *p* < 0.001), respectively. While, the presence of C/C genotype in STAT6 rs167769 was inversely associated with HCC development (OR = 0.063, 95% CI = 0.013–0.308, *p* = 0.001). This model showed strong predictive capacity with area under the ROC curve 0.948 (95% CI = 0.903–0.993) and 87.5% specificity, 89.5% accuracy, and 90.9% sensitivity. This highlights the important role played by IL-13/STAT6 axis in cancer development in NASH, in both lower and advanced fibrosis grades patients.

## 4. Discussion

Obesogenic cancers are currently at the top of public health concern. Accounting for 40% of cancer diagnoses in the United States, obesity and being overweight are now recorded as significant contributing factors to cancer incidences and death [36]. Being historically related to viral hepatitis, liver cancer becomes markedly alarmed by NASH with the introduction of novel accessible antiviral treatments [37].

Despite a consistent relationship between NASH and HCC development, no approved pharmacological therapy has yet been introduced. This challenges researchers to tackle the exact mechanisms for understanding disease pathogenesis and progression. Recently, attention has been raised to type 2 immunity and its crucial contributions to pathological fibrosis and cancer development in multiple organs [7]. IL-13, a type 2 immunity effector, is directly involved in tissue repair and regeneration following injury; however, when persistently activated, it transforms these tissue-regenerative responses into progressive fibrotic disorder with unknown clear mechanism or signaling [38]. Accordingly, this research aimed to address and underscore the role of IL-13, as well as its receptors and some of the signaling genes and integrated crosstalk genes, in the prediction of advanced fibrosis progression and HCC development in NASH. 

This study comparatively investigated well-characterized male NASH patients with different fibrosis grades and NASH-HCC patients. This study found a significant direct association between higher serum IL-13 levels and cancer progression in NASH. Interestingly, recent reports proved that IL-13 may play a crucial role in other tumors. Whereas, IL-13 was proved to be associated with obesity-related colorectal tumorigenesis [39]. Inflammation provoked by obesity notably increased expression of IL-13, which in turn leads to increased expression of IL-13R1, ending up with activation of downstream phosphorylation of the transcription factor STAT6, suggesting a possible mechanism of carcinogenesis in colon cancer [40]. Our study agreed with this report, as higher serum IL-13 and polymorphism at STAT6 rs167769 were reported to be the strongest variables associated with cancer progression in NASH patients, regardless of their fibrosis stages. In accordance, evidence has suggested that STAT6 is involved in the HCC process and may predict a worse prognosis in patients with HCC [41]. However, this study specifically introduced the C/C genotype carrier in the STAT6 rs167769 gene as a predictive marker with a strong inverse relation to HCC development in NASH patients, irrespective of their fibrosis stage. More interestingly, higher IL-13 levels and the absence of the STAT6 rs167769 C/C genotype also significantly predict HCC development in NASH when compared to low F1 and F2 patients, and advanced fibrosis grades, F3 and F4 patients. This confirms that IL-13/STAT6 plays a major role in HCC development in NASH. The underlined mechanism for STAT6 rs167769 polymorphism is still unclear. Whereas, some studies showed that the T alleles of STAT6 rs167769 could increase STAT6 promoter activity, which further increases the STAT6 signaling axis in dermatitis [42]. Hence, it is suggested that C allele has a protective role via decreasing STAT6 signaling activation. Other studies confirmed that SNP at STAT6 rs167769 is strongly associated with STAT6 expression in the blood and lungs [43]. More interestingly, previous reports suggest that STAT6 rs167769 SNP is significantly associated with immune function via decreasing interferon gamma (IFNγ) production [44]. Notably, it was previously found that diets rich in fat significantly suppress natural killer T (NKT) cell-derived IFNγ production, but significantly enhance the production of IL-13, which activates carcinogenesis in NASH [45]. Another possible explanation for our finding of STAT6 association with HCC development in NASH was supported by a recent study, which showed that variant of STAT6 rs167769 is strongly associated with eosinophilic esophagitis [46]. Interestingly, it was confirmed recently that progression of NASH was significantly associated with increasing eosinophilic type 2 liver inflammation in experimental, as well as in human patient biopsies [6]. All these evidences provided a possible explanation of our finding of IL-13/STAT6 rs167769 association with HCC in NASH. However, functional analysis studies are required to understand the exact mechanism of action.

Interestingly, this study found a significant association of the hippo signaling pathway represented through YAP1, a downstream effector of hippo signaling, with HCC development in NASH patients. This study showed that C allele carriers in YAP1 rs11225163 are protected from HCC development in NASH. The hippo signaling seems to be the ideal candidate pathway that governs hepatocyte proliferation during regeneration [47]. Our results are in alignment with a recent report, which proved that progression HCC in NASH was attributed to YAP1 [48]. Surprisingly, this study sheds light on the possible integration of IL-13/STAT6 axis and Hippo signaling through YAP1 in cancer progression in NASH. In accordance, it has been recently proven that YAP1 expression is differentially regulated by IL-13, which, upon STAT6 activation, enhances the expression of the M2- macrophage associated genes, which in turn promotes tissue repair and control inflammation in inflammatory bowel syndrome [49]. However, future studies are required to confirm our findings for HCC development in NASH patients.

It is important to mention that by classifying NASH patients into low F1 and F2 fibrosis grades and high F3 and F4 fibrosis grades, C allele carriers in YAP1 rs11225163 lost its significant association with HCC development in NASH. This finding might be explained by the absence of cirrhosis among NASH patients. On the other side, 90.9% of NASH-HCC were cirrhotic. Interestingly, 66.6% of the T/T genotype in YAP1 rs11225163 were cirrhotic NASH-HCC patients. While, only 33.3% of the T/T genotype in the YAP1 rs11225163 gene were in NASH patients. Upon classifying NASH into lower (F1 and F2) and higher grades (F3 and F4), all T/T genotype in the YAP1 rs11225163 gene were of higher fibrosis grades (F3 and F4). This highlights that YAP1 rs11225163 might be associated with cirrhosis in NASH-HCC. However, the sample size decreased significantly upon subclassification of NASH patients into low and high fibrosis grades. That is why YAP1 rs11225163 polymorphism lost its association with cancer upon subclassification. However, this finding might indicate that YAP1 might lie at the intercept between severe advanced fibrosis/cirrhosis and HCC development in NASH. In agreement, previous studies showed that YAP activation in HSCs and hepatocytes could possibly increase carcinogenesis in cirrhotic livers [47,50]. However, a larger sample size is required to confirm this suggestion.

This study found a significant contribution of IL-13 rs20541, IL-13R2 rs5946040, and PD-L1 rs2282055 at univariate analysis with cancer progression in NASH. However, the contribution of IL-13/STAT6 and YAP1, together with serum AFP, are proven to have the upper hand in predicting cancer development in NASH. In fact, serum AFP is a well-known classical marker for HCC [51]. Its level was strongly proved to be associated with cancer progression in NASH, as highlighted by this study. However, combining IL-13/STAT6 and YAP1 increases the sensitivity and accuracy of serum AFP, which again highlights the critical role played by variables in predicting HCC development in NASH. Additionally, combining IL-13/STAT6 with serum AFP also increased predictive power of serum AFP and increased its specificity, sensitivity, and accuracy for predicting HCC development in high fibrosis grades F3 and F4 NASH patients. In addition, combining IL-13/STAT6 with serum AFP also increased predictive capacity of serum AFP and increased its sensitivity and accuracy in predicting HCC development in low fibrosis grades, F1 and F2 NASH patients. 

Interestingly, this study highlighted the importance of formulating a predictive model for HCC development in the absence of higher fibrosis grades in NASH. Whereas, a significant association was found between higher serum AFP and higher serum IL-13 levels with HCC development in F1 and F2 NASH patients. While, the presence of C/C genotype in STAT6 rs167769 was inversely associated with HCC development in F1 and F2 NASH patients. This predictive model showed excellent predictive capacity improving the predictive capacity of serum AFP alone. It is important to note that all NASH-HCC patients in this study were cirrhotic or with advanced fibrosis grades. It agreed with a previous study predicting HCC development in NASH [52]; however, previous studies reported that HCC can develop de novo in patients with NASH without the presence of cirrhosis [53,54]. Therefore, this study might aid in understanding the mechanism of progression of HCC in absence of fibrosis grades in the future.

The second aim of this study was to investigate the possible role played by our studied variables for fibrosis progression in NASH. Interestingly, this study showed that higher serum IL-13 is significantly associated with advanced fibrosis grades in NASH. This comes in alignment with previous reports, which showed the disturbed healing process in NASH contributed to fibrosis progression via chronic activation of type 2 inflammation manifested by increased serum IL-13 levels [9,10]. It was explained by overproduction of Th2 cytokines, which typically promote B-cell hyperactivity and humoral immune responses, whereas T cell hyperactivity and inflammation frequently associated with an excess of Th1 and Th17 cytokines at earlier stages of the diseases [55]. However, IL-4/IL-13-secreting invariant nature killer T cells (iNKT) were prevalent later in the disease [56].

Additionally, this study also highlighted the contribution of PD-L2 C allele carriers together with serum levels of IL-13 for advanced fibrosis progression in NASH. There are a multitude of mechanisms that dampen hepatic immunity upon hepatocyte inflammation due to abnormal lipid accumulation and peroxidation in NASH. Of these lies the involvement of NKT cells at the center of interest, by limiting T cell responses and inducing the upregulation of the inhibitory molecules PD-L1 and PD-L2 on dendritic cells (DC) [57]. This interplay among innate lymphocyte populations switches the convert of a quiescent immune environment into a cellular battlefield during chronic inflammatory diseases, such as NASH [58]. Thus, this study adds evidence for the possible involvement of PD-L2 and IL-13 in advanced fibrosis progression in NASH.

In summary, this study reports a striking association between serum levels of IL-13 and SNP at PD-L2 rs7854413 with advanced fibrosis in NASH patients, independent with other variables. This study uniquely formulated a predictive model with moderate predictive capacity for advanced fibrosis progression in NASH. Additionally, this study showed a significant association between serum levels of IL-13, serum AFP levels, and SNPs at STAT6 rs167769 and YAP1 rs11225163 with HCC development in NASH. Serum levels of IL-13, serum AFP levels, and SNPs at STAT6 rs167769 are associated with HCC development when compared to lower and higher fibrosis grades in NASH patients. These predictive models significantly increased the predictive capacity of serum AFP for HCC prediction in NASH. These data highlight the importance of understanding the contribution of IL-13 and its integrating and signaling genes in NASH-HCC pathogenesis, and if validated, may contribute to a tailored, personalized approach to cost effective surveillance and detection, possibly targeting NASH-HCC patients. Therefore, this study strongly recommends further investigation using a multi-racial validation cohort, required to explore clinical significance. Additionally, functional studies are required to investigate the mechanisms of PD-L2/IL-13/STAT6 and YAP1 in fibrosis progression and HCC development in NASH.

## 5. Conclusions

Stratifying patients based on their genetic background is a reliable cornerstone approach for better understanding and targeting of diseases, especially multidisciplinary diseases with complex pathophysiology and integrated signaling crosstalk, such as NASH. This study sheds light on the possible integration of different pathways in fibrosis progression and HCC development in NASH. Finally, this study adds clinical proof for the important role that is played by type 2 immunity and the aberrant healing process for advanced fibrosis progression and HCC development in NASH.

## Figures and Tables

**Table 1:** Baseline clinical characteristics of studied patients included in the present study.

Characteristics	NASH (n = 79)	NASH-HCC (n = 55)	*p*-Value	Statistics (Univariate) OR (95%CI)
Age mean ± SD (years)	61.2 ± 5.9	63.9 ± 6.4	0.017	1.073 (1.012–1.138)
BMI, median (range), kg/m^2^	29 (4.3)	28.3 (7.3)	-	
^a^ AST median (range), U/L	53 (35)	67 (18)	-	
^b^ ALT median (range), U/L	48 (21)	44 (11)	-	
Albumin median (range), g/dL	3.7 (0.5)	3.3 (0.7)	-	
Bilirubin median (range), mg/dL	1.5 (0.8)	1.3 (0.7)	-	
Hemoglobin median (range), g/dL	13.4 (2.7)	12 (1.7)	-	
Platelets median (range), 10^9^/L	178 (84)	129 (97)	-	
^c^ TLC median (range), 10^9^/µL	6.5 (2.7)	6 (1.4)	-	
^d^ TAG median (range), mg/dL	158 (60)	160 (47)	-	
Glucose median (range), mg/dL	101 (18)	106 (22)	-	
Insulin median (range), µU/mL	11.6 (3.3)	12 (3.7)	-	
^e^ HOMA-IR median (range)	3 (0.9)	3.2 (1)	-	
^f^ AFP median (range), ng/mL	9.5 (5)	407.7 (570)	<0.001	7.641 (3.6–16.18)
IL-13 mean ± SD, (ng/L)	4.9 ± 1.7	7.85 ± 4.3	<0.001	1.616 (1.3–2.008)
Fibrosis stage, [n(%)]			<0.001	
F1–F2	41 (51.8)	-	-	
F3–F4	38 (48.1)	5 (9)	-	
Cirrhosis	-	50 (90.9)	-	

a AST, aspartate aminotransferase; b ALT, alanine aminotransferase; c TLC, total leukocyte count; d TAG, triacylglycerol, e HOMA-IR, homeostatic model assessment of insulin resistance; f AFP, alpha-fetoprotein; NASH: non-alcoholic steatohepatitis; HCC: hepatocellular carcinoma; IL-: interleukin; n, number of samples; OR, odds ratio; 95%CI Confidence Interval.; Simple binary logistic regression analysis was performed.

**Table 2:** Association of genes polymorphisms with cancer development in NASH.

Genotype/Allele		NASH (n = 79)	NASH-HCC (n = 55)	Chi^2^ *p-*value	OR (95% CI)
IL-13 rs20541 [n(%)]				Chi^2^ = 12.12, *p* = 0.002	
	A/A	3 (3.7)	12 (21.8)		
	A/G	30 (37.9)	12 (21.8)		
	G/G	46 (58.2)	31 (56.3)	chi^2^ = 10.5 0.004	7.07 (1.89–26.44)
IL-13R1 rs2248841 [n(%)]				Chi^2^ = 1.854, *p* = 0.396	
	C/C	6 (7.5)	8 (14.5)		
	T/C	18 (22.7)	10 (18.1)		
	T/T	55 (69.6)	37 (67.2)		
IL-13R2 rs5946040 [n(%)]				Chi^2^ = 6.09, *p* = 0.048	
	G/G	9 (11.3)	14 (25.4)		
	G/T	6 (7.5)	1 (1.8)		
	T/T	64 (81)	40 (72.7)	chi^2^ = 4.5 0.038	2.656 (1.056–6.677)
STAT6 rs167769 [n(%)]				Chi^2^ = 24.4, *p* < 0.001	
	C/C	70 (88.6)	29 (52.7)	chi^2^ = 21.6 <0.001	0.124 (0.052–0.297)
	C/T	6 (7.5)	24 (43.6)		
	T/T	3 (3.7)	2 (3.6)		
YAP1 rs11225163 [n(%)]				Chi^2^ = 6.14, *p* = 0.046	
	C/C	39 (49.3)	20 (36.3)		
	C/T	34 (43)	23 (41.8)		
	T/T	6 (7.5)	12 (21.8)		
C allele carriers/C allele non carriers		73/6	43/12	chi^2^ = 5.56 0.022	0.295 (0.103–0.842)
PD-L1 rs2282055 [n(%)]				Chi^2^ = 6.273, *p* = 0.043	
	G/G	5 (6.3)	11 (2)		
	G/T	12 (15.1)	5 (9)		
	T/T	62 (78)	39 (70.9)	chi^2^ = 5.76 0.022	3.7 (1.206–11.352)
PD-L2 rs7854413 [n(%)]				Chi^2^ = 1.99, *p* = 0.37	
	C/C	5 (6.3)	6 (10.9)		
	T/C	23 (29.1)	11 (20)		
	T/T	51 (64.5)	38 (69)		

OR, odds ratio; 95%CI Confidence Interval; NASH: non-alcoholic steatohepatitis; HCC: hepatocellular carcinoma; IL-: interleukin; YAP: Yes-associated protein; STAT6: signal transducer and activator of transcription 6; PD-L: programmed death ligand, chi-square (chi^2^) test for genotype distribution was performed.

**Table 3:** Association between adjusted significant parameters with HCC development in NASH.

Parameters	Univariate Regression Analysis		Multivariate Regression Analysis	
	Adjusted OR (95%CI)	*p*-value	Adjusted OR (95%CI)	*p*-value
^*^ AFP ng/mL	7.854 (3.58–17.2)	<0.001	19.6 (4.36–88.85)	<0.001
IL-13 ng/L	1.727 (1.368–2.18)	<0.001	1.9 (1.211–2.99)	0.005
IL-13 rs20541, G/G genotype	6.172 (1.622–23.47)	0.008	-	0.491
IL-13R2 rs5946040, T/T genotype	3.5 (1.301–9.445)	0.013	-	0.832
STAT6 rs167769, C/C genotype	0.092 (0.035–0.244)	<0.001	0.015 (0.002–0.123)	<0.001
YAP1 rs11225163, C allele carriers/C allele non carriers	0.213 (0.07–0.647)	0.006	0.047 (0.006–0.386)	0.004
PD-L1 rs2282055 T/T genotype	4.015 (1.272–12.669)	0.018	-	0.598

NASH: non-alcoholic steatohepatitis; HCC: hepatocellular carcinoma; IL-: interleukin; YAP: Yes-associated protein; STAT6: signal transducer and activator of transcription 6; PD-L: programmed death ligand. * logarithmically transformed data. The statistical analysis was performed using general linear modeling for adjusting significant covariate (age), then multiple stepwise logistic regression analysis was performed.

**Table 4:** Association of gene polymorphisms with advanced fibrosis grades in NASH patients.

Genotype/Allele		NASH F1–F2 (n = 40)	NASH F3–F4 (n = 39)	Chi^2^ *p*-value	OR (95% CI)
IL-13 rs20541 [n(%)]				Chi^2^ = 9.963, *p* = 0.007	
	A/A	3 (7.5)	-		
	A/G	9 (22.5)	21 (53.8)		
	G/G	28 (70)	18 (46.1)		
A allele carriers/A allele non carriers		12/40	21/39	chi^2^ = 4.617 0.034	2.722 (1.081–6.6858)
IL-13R1 rs2248841 [n(%)]				Chi^2^ = 2.879, *p* = 0.237	
	C/C	3 (7.5)	3 (7.7)		
	T/C	6 (15)	12 (30.7)		
	T/T	31 (77.5)	24 (61.5)		
IL-13R2 rs5946040 [n(%)]				Chi^2^ = 7.89, *p* = 0.018	
	G/G	6 (15)	3 (7.6)		
	G/T	6 (15)			
	T/T	28 (70)	36 (92.3)		
G allele carriers/G allele non carriers		12/40	3/39	chi^2^ = 6.388 0.018	0.194 (0.05–0.756)
STAT6 rs167769 [n(%)]				Chi^2^ = 1.045, *p* = 0.593	
	C/C	34 (85)	36 (92)		
	C/T	4 (10)	2 (5.1)		
	T/T	2 (5)	1 (2.5)		
YAP1 rs11225163 [n(%)]				Chi^2^ = 6.69, *p* = 0.127	
	C/C	21 (52.5)	18 (46.1)		
	C/T	19 (47.5)	15 (38.4)		
	T/T		6 (15.3)		
PD-L1 rs2282055 [n(%)]				Chi^2^ = 0.252, *p* = 0.882	
	G/G	2 (5)	3 (7.6)		
	G/T	6 (15)	6 (15.3)		
	T/T	32 (80)	30 (37.9)		
PD-L2 rs7854413 [n(%)]				Chi^2^ = 12.923, *p* = 0.002	
	C/C		5 (12.8)		
	T/C	7 (17.5)	16 (41)		
	T/T	33 (82.5)	18 (46.1)		
C allele carriers/C allele non carriers		7/40	21/39	chi^2^ = 7.495 0.001	5.5 (1.963–115.411)

OR, odds ratio; 95%CI Confidence Interval; NASH: non-alcoholic steatohepatitis; HCC: hepatocellular carcinoma; IL-: interleukin; YAP: Yes-associated protein; STAT6: signal transducer and activator of transcription 6; PD-L: programmed death ligand. Chi-square test for genotype distribution was performed.

**Table 5:** Simple and multiple regression analysis for advanced fibrosis progression in NASH patients.

Parameters	Univariate Regression Analysis		Multivariate Regression Analysis	
	OR (95%CI)	*p*-value	OR (95%CI)	*p*-value
IL-13 ng/L	1.496 (1.111–2.014)	0.008	1.432 (1.022–2.008)	0.037
IL-13 rs20541, A allele carriers/A allele non carriers	2.722 (1.081–6.685)	0.034	-	0.118
IL-13R2 rs5946040, G allele carriers/G allele non carriers	0.194 (0.05–0.756)	0.018	-	0.072
PD-L2 rs7854413, C allele carriers/C allele non carriers	5.5 (1.963–115.411)	0.001	3.797 (1.216–11.875)	0.022

NASH: non-alcoholic steatohepatitis; HCC: hepatocellular carcinoma; IL-: interleukin; PD-L: programmed death ligand. The statistical analysis was performed using multiple stepwise logistic regression analysis was performed.

## Abbreviations

Non-alcoholic steatohepatitis (NASH), hepatocellular carcinoma (HCC), single nucleotide polymorphisms (SNPs), alpha-fetoprotein (AFP), area under the operating curve (AUROC), complete blood picture (CBC), serum transaminases (ALT, AST), real-time polymerase chain reaction (RT-PCR), enzyme-linked immunosorbent assay (ELISA), homeostatic model assessment of insulin resistance (HOMA-IR), interleukin (IL), receiving operating curve (ROC). Interleukin 13 (IL-13), Interleukin 13 receptor (IL-13R), signal transducer and activator of transcription (STAT6), yes-associated protein (YAP1), programmed death-ligand (PD-L).
